# Procedural safety of rotational atherectomy and modified balloon angioplasty: insights from a German national registry

**DOI:** 10.1007/s00392-024-02538-8

**Published:** 2024-09-11

**Authors:** Alexander Maier, Mark Colin Gissler, Markus Jäckel, Vera Oettinger, Lucas Bacmeister, Adrian Heidenreich, Jonathan Rilinger, Lukas A. Heger, István Bojti, Christian Weber, Dennis Wolf, Ingo Hilgendorf, Faridun Rahimi, Miroslaw Ferenc, Dirk Westermann, Klaus Kaier, Constantin von zur Mühlen

**Affiliations:** 1 Department of Cardiology and Angiology, University Heart Center Freiburg—Bad Krozingen, Faculty of Medicine, University of Freiburg, Freiburg, Germany; 2 Institute of Medical Biometry and Statistics, Faculty of Medicine, University of Freiburg, Freiburg, Germany

**Keywords:** Rotational atherectomy, Modified balloon, Cutting balloon, Scoring balloon, Coronary artery disease, In-hospital safety, Procedural safety, Real-world cohort

## Abstract

**Background:**

Modified balloons (MB) and rotational atherectomy (RA) are recommended tools for treatment of coronary plaques with superficial calcium. Knowledge about in-hospital safety is limited.

**Methods:**

Patients with coronary artery disease who underwent coronary angiography with RA or MB angioplasty in Germany were identified via ICD and OPS codes from 2017 to 2020. Acute coronary syndromes were excluded. Since patients were not randomized toward MB or RA, potential confounding factors were taken into account using the propensity score methods. Thereby, inverse probability weighting was applied.

**Results:**

Ten thousand.ninety-twopatients underwent RA with an increasing trend from 1817 in 2017 toward 3166 in 2020. MBs were used in 22,378 patients also with an increasing trend from 4771 in 2017 toward 6078 in 2020.

Patients receiving RA were older (74.23 ± 8.68 vs. 71.86 ± 10.02, *p* < 0.001), had a higher Charlson Comorbidity Index (2.07 ± 1.75 vs. 1.99 ± 1.76, *p* = 0.001) and more frequently left main (17.96% vs. 12.91%, p < 0.001) or three vessel disease (66.25% vs. 58.10%, *p* < 0.001). Adjusted procedural risk of major adverse cardiac and cerebrovascular events (MACCE) was similar in both groups, while pericardial effusion (RR 2.69; 95% CI 1.88–3.86, *p* < 0.001), pericardial puncture/pericardiotomy/pericardial tamponade (RR 2.66; 95% CI 1.85–3.81, *p* < 0.001) and bleeding (RR 1.65; 95% CI 1.12–2.43, *p* < 0.011) occurred more frequently in patients receiving RA. Patients treated with RA at high volume centers were hospitalized shorter (*p* = 0.005) and had a lower rate of acute cerebrovascular events (*p* < 0.001). Rate of MACCE, bleeding and pericardial puncture were not influenced by the annual RA numbers per center.

**Conclusion:**

MBs had a lower risk of bleeding and pericardial puncture. Patients treated at centers with high annual RA procedure numbers had a lower risk of acute cerebrovascular events and were hospitalized shorter.

**Supplementary Information:**

The online version contains supplementary material available at 10.1007/s00392-024-02538-8.

## Introduction

Relevant coronary artery calcification (CAC) is present in 20–30% of patients with coronary artery disease (CAD) [1–3], [4] and is associated with unfavorable outcomes after percutaneous coronary interventions (PCI) [2, 3, 5–7]. Clinical management of severely calcified coronary stenoses is challenging and requires the careful selection of dedicated interventional devices based on the anatomy of the target lesion and its underlying calcium morphology [8]. The EAPCI clinical consensus statement in collaboration with the EURO4C-PCR group recommends a step-wise management for patients with heavily calcified coronary stenosis, starting with a thorough imaging assessment of calcified lesions and the corresponding selection of appropriate interventional tools for lesion preparation [9].

Within the last decade, modified balloon-based as well as atherectomy-based-techniques have been increasingly recognized as valuable tools for preparation of calcified coronary lesions during PCI. Modified balloons (MB) allow for a more effective dilatation through focal concentrations of dilating force on cutting or scoring elements of the balloon surface. The cutting balloon (Wolverine Cutting Balloon, Boston Scientific) is a non-compliant balloon with three or four microsurgical blades (atherotomes) mounted longitudinally on its outer surface. Scoring balloons are semi-compliant (AngioSculpt, Philips; NSE Alpha, Braun) or non-compliant (ScoreFlex NC, OrbusNeich) balloons surrounded by external nitinol spiral scoring wires on their surface. Rotational atherectomy (RA, Rotablator System, Boston Scientific) employs an elliptically shaped metallic burr with a diamond-coated distal tip rotating with a recommended rotablation speed between 135 000 and 180 000 rpm that preferentially ablates inelastic tissue, such as calcified plaque material [10].

If moderate to severe calcification is identified in a target lesion based on angiography or coronary computed tomography angiography, according to the current consensus direct stenting should be avoided and crossability of the lesion should be assessed by a small balloon [9]. While the use of cutting/scoring balloons is reserved for balloon-crossable lesions, RA can be employed to lesions, which are accessible for a rotawire (a specialized 0.009 inch guidewire) or microcatheter [4, 9]. Both are recommended options for lesions with superficial calcium [9].

Studies focusing on procedural success demonstrated that cutting balloon angioplasty allows for achieving significantly larger acute lumen gain at lower inflating pressure, especially in aorto-ostial lesions and lesions with evidence of dissections in comparison to conventional balloon pre-dilatation [11–15]. Similarly, pretreatment with a scoring balloon has been demonstrated to improve final stent expansion compared with conventional balloon angioplasty [16, 17]. Conversely, RA prior to drug eluting stent (DES) implantation can improve the immediate procedural success rate compared to direct stenting or pre-dilatation with modifying balloon angioplasty [18–21].

However, real-world data about the in-hospital safety of RA or MB techniques are scarce. In this study we therefore analyzed patients’ characteristics and procedural safety of cutting or scoring balloon angioplasty and RA from a German nationwide registry. In-hospital safety parameters for both procedures were compared by a propensity score approach. In addition, we evaluated whether outcomes were dependent on procedure numbers per year performed by a single center.

## Methods

### Data source

Anonymized and aggregated inpatient data were obtained from the German nationwide inpatient sample via the Research Data Center of the Germany’s Federal Bureau of Statistics as previously described [22, 23]. Since our study did neither require nor involve access to individual patient records, ethics committee approval and informed consent were not required in accordance with German law.

### Diagnoses and outcomes definitions

We requested data and numbers of patients with coronary heart disease (ICD-10 code I25.11, I25.12, I25.13, I25.14 as main or secondary diagnosis) that underwent planed coronary angiography (German Procedure Classification/OPS code 1–275) with cutting or scoring balloon angioplasty (8–837.q) and RA (8–837.5) for each year from 2017 to 2020 from the German Research Data Center. Patients with acute coronary syndromes such as NSTEMI, STEMI or unstable angina were excluded from the dataset.

The following patient baseline characteristics were requested: Age, female sex, Charlson comorbidity index (see [24] for complete ICD-10 list), heart failure NYHA III or IV (I5013 and I5014) arterial hypertension (I10), coronary artery disease (I25), coronary-one-vessel disease (I2511), coronary-two-vessel disease (I2512), coronary-three-vessel disease (I2513), left main disease (I2514), in-stent-stenosis (I2516), previous myocardial infarction (I252), previous cardiac surgery (Z951 2 3 4), atrial fibrillation (I480 1 2 9), peripheral vascular disease (I702 8 9 I739), carotid disease (I652), chronic obstructive lung disease (J44), pulmonary hypertension (I27), chronic renal disease (N18), diabetes (E10 1 2 3 4) and previous stroke (I69*). The CHA2DS2-VASc score was calculated as described elsewhere [22]. Congestive heart failure, hemiplegia or paraplegia, dementia, connective tissue disease, peptic ulcera disease, mild liver disease, moderate to severe liver disease, cancer (ICD C*) and metastatic solid tumor are components of the Charlson comorbidity index [24].

The following in-hospital outcome parameters were requested from 2017 to 2020: MACCE (composed of in-hospital mortality, myocardial infarction (I21) and stroke (I63)), acute cerebrovascular events (one of 3–200; 3–220; 3–800; 3–820; 8–981.x; I60.x; I61.x; I63.x; I64 had to be fullfilled), pericardial effusion (I312 or I313), pericardial drainage (composed of pericardial tamponade, pericardial puncture or pericardiotomy (OPS 1–842* 5–370.* 8–152.0* 5–374.*)), bleeding (transfusion > 5 red blood cell concentrates, 8–800.c1–8–800.cr), deep vein thrombosis (I80), stent implantantion (OPS 8837 m*). Length of hospital stay and in-hospital mortality are part of DESTATIS’ main set of variables.

### Statistical methods

Categorical variables were presented as n (%). Continuous variables were summarized as mean ± SD. Pearson’s Chi-square and t tests were used to make descriptive comparisons between groups as appropriate.

Since patients were not randomized towards MB or RA, potential confounding factors were taken into account using the propensity score methods. Thereby, inverse probability weighting was applied. The propensity score is defined as the conditional probability of an individual for being in the treatment group, given a group of observed covariates. For the propensity score estimation, we fit a logistic regression model controlling for 30 predetermined covariates (all variables listed in Table 1). For the outcome models, we chose Poisson-regression models for the inverse probability weighting approach. Thereby, cluster-robust standard errors were used to account for the correlation of error terms of patients treated in the same hospital. Exponentiated coefficients of these weighted Poisson-regression models may be interpreted as relative risks in case of dichotomous endpoints or incidence rate ratios in case of continuous endpoints (length of stay). To determine the impact of procedure volumes on the endpoints length of stay, MACCE, acute cerebrovascular events, pericardial effusion, pericardial drainage and bleeding, multivariable Poisson-regression analyses were performed. For risk adjustment, age, the Charlson Comorbidity Index and procedure volume were included as continuous covariates while all categorical characteristics listed in Table 1 were included as categorical covariates. Cluster-robust standard errors were used to account for the correlation of error terms of patients treated in the same hospital.

**Table 1:** Baseline charateristics of patients undergoing rotational atherectomy or scoring/cutting balloon procedures in Germany between 2017 and 2020

Characteristic	Rotational atherectomy *n* = 10,092	modified balloon *n* = 22,378	*p*
Women	2278 (22.58%)	*5428 (24.26%)*	** < *0.001***
Age	*74.23* ± *8.68*	71.86 ± 10.02	** < *0.001***
Charlson Comorbidity Index (CCI)	*2.07* ± *1.75*	1.99 ± 1.76	** < *0.001***
Congestive heart failure	4295 (42.56%)	9273 (41.44%)	0.058
NYHA III or IV	*2267 (22.46%)*	4738 (21.17%)	***0.009***
Arterial hypertension	*8502 (84.24%)*	18,278 (81.72%)	** < *0.001***
Coronary artery disease	10,050 (99.58%)	22,253 (99.44%)	0.097
Coronary 1-vessel disease	953 (9.44%)	*3074 (13.74%)*	** < *0.001***
Coronary 2-vessel disease	2,412 (23.90%)	*6,055 (27.06%)*	** < *0.001***
Coronary 3-vessel disease	*6686 (66.25%)*	13,001 (58.10%)	** < *0.001***
Left main disease	*1813 (17.96%)*	2888 (12.91%)	** < *0.001***
In-stent stenosis	422 (4.18%)	*4705 (21.03%)*	** < *0.001***
Previous myocardial infarction	1651 (16.36%)	*4521 (20.20%)*	** < *0.001***
Previous cardiac surgery	*1458 (14.45%)*	2495 (11.15%)	** < *0.001***
Atrial fibrillation	*2637 (26.13%)*	5288 (23.63%)	** < *0.001***
CHA_2_DS_2_ VASC (mean ± SD)	*4.29* ± *1.27*	4.08 ± 1.36	** < *0.001***
Peripheral vascular disease	*1,251 (12.40%)*	2,169 (9.69%)	** < *0.001***
Carotid disease	319 (3.16%)	539 (2.41%)	** < *0.001***
COPD	845 (8.37%)	1799 (8.04%)	0.309
Pulmonary hypertension	588 (5.83%)	1330 (5.94%)	0.679
Chronic renal disease	*2612 (25.88%)*	5378 (24.03%)	** < *0.001***
Diabetes	*3773 (37.39%)*	7962 (35.58%)	***0.002***
Previous stroke	110 (1.09%)	196 (0.88%)	0.065
Hemiplegia or paraplegia	*20 (0.20%)*	22 (0.10%)	***0.021***
Dementia	*104 (1.03%)*	172 (0.77%)	***0.017***
Connective tissue disease	103 (1.02%)	*304 (1.36%)*	***0.011***
Peptic ulcer disease	32 (0.32%)	70 (0.31%)	0.949
Mild liver disease	112 (1.11%)	269 (1.20%)	0.475
Moderate to severe liver disease	18 (0.18%)	35 (0.16%)	0.650
Cancer	135 (1.34%)	287 (1.28%)	0.685
Metastatic solid tumor	17 (0.17%)	52 (0.23%)	0.247

Two-sided p values are given, and statistical significance was considered as *p*-value < 0.05. No adjustments for multiple testing were done. All analyses were carried out using Stata 17 (StataCorp, College Station, Texas, USA).

## Results

### Annual procedure numbers for rotational atherectomy and modified balloon angioplasty

**Fig. 1:**
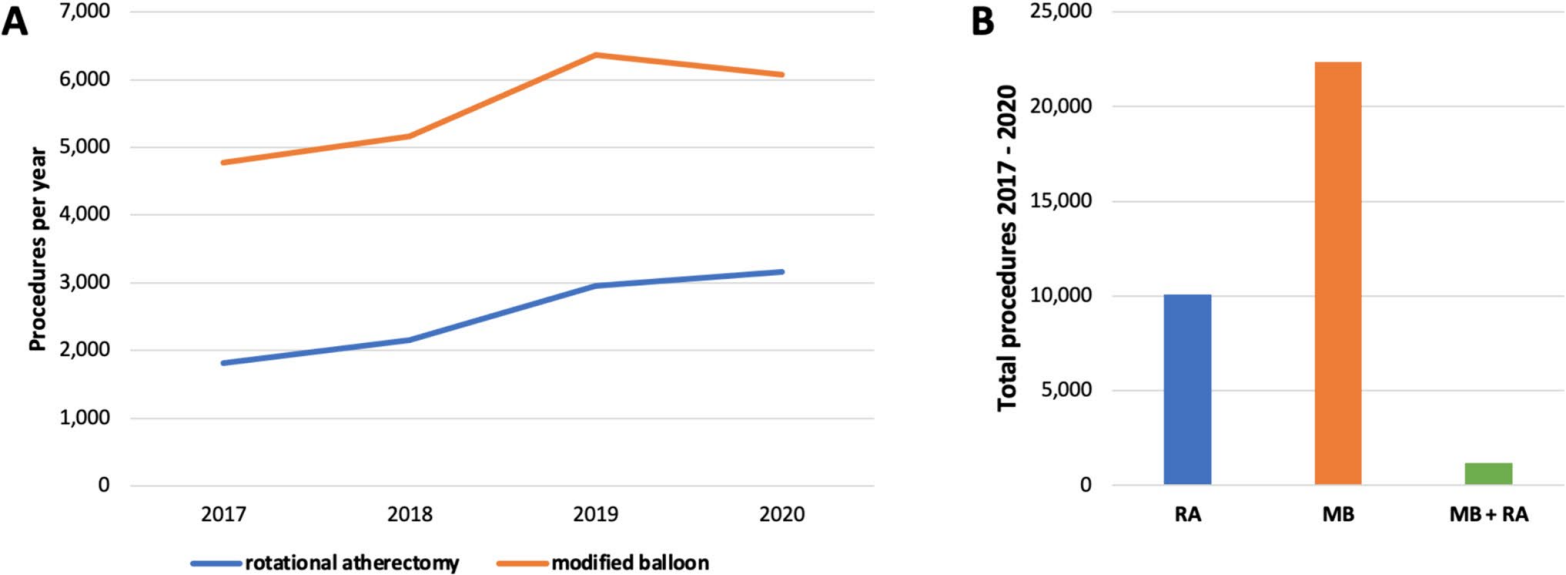
Annual numbers of rotational atherectomy or scoring/cutting balloon procedures in Germany from 2017 to 2020 (**A**) and total number of procedures (**B**)

RA was done in 0.51% and MB angioplasty in 1.14% of all planed coronary angiographies in Germany from 2017 to 2020. Procedure numbers for RA and MB angioplasty both increased from 2017 to 2020. In 2017 1817 RA procedures and 4771 scoring/cutting balloon angioplasties were performed, which increased in 2020 to 3166 for RA and 6078 for cutting/scoring balloons, respectively (Fig. 1A). In total, 10,092 RA procedures and 22,378 MB angioplasty procedures were performed from 2017 to 2020 in Germany. 1179 patients underwent both procedures during their hospitalization in this period (Fig. 1B).

### Patient characteristics

Characteristics of patients receiving RA or MB angioplasty are given in Table 1. Patients receiving RA were older (74.23 ± 8.68 vs. 71.86 ± 10.02, *p* < 0.001), had a higher Charlson Comorbidity Index (2.07 ± 1.75 vs. 1.99 ± 1.76, *p* < 0.001), more frequently arterial hypertension (84.24% vs. 81.72%, *p* < 0.001), coronary three vessel disease (66.25% vs. 13,001 58.10%, *p* < 0.001) and left main disease (17.96% vs. 12.91%, *p* < 0.001). They have more often undergone previous cardiac surgery (14.45% vs. 11.15%, *p* < 0.001) and had more often atrial fibrillation (26.13% vs. 23.63%, p < 0.001) with a higher CHA_2_DS_2_-VASc score (4.29 ± 1.27 vs. 4.08 ± 1.36, *p* < 0.001). Peripheral vascular disease (12.40%) vs. 9.69%, *p* < 0.001), chronic renal disease (25.88% vs. 24.03%, *p* < 0.001) and diabetes (37.39% vs. 35.58%, *p* = 0.002) were also prevalent more often in the RA group. Congestive heart failure was distributed equally between the groups, while heart failure NYHA III or IV was more frequently in the RA group (22.46% vs. 21.17%, *p* = 0.009). Previous myocardial infarction was more common in patients with MB angioplasty (20.20% vs. 16.36%, *p* < 0.001) and previous stroke did not show a significant difference between the two groups. Taken together, individuals undergone RA had a more severe cardiovascular disease profile compared with patients receiving MB angioplasty.

### Procedural safety comparison

**Fig. 2:**
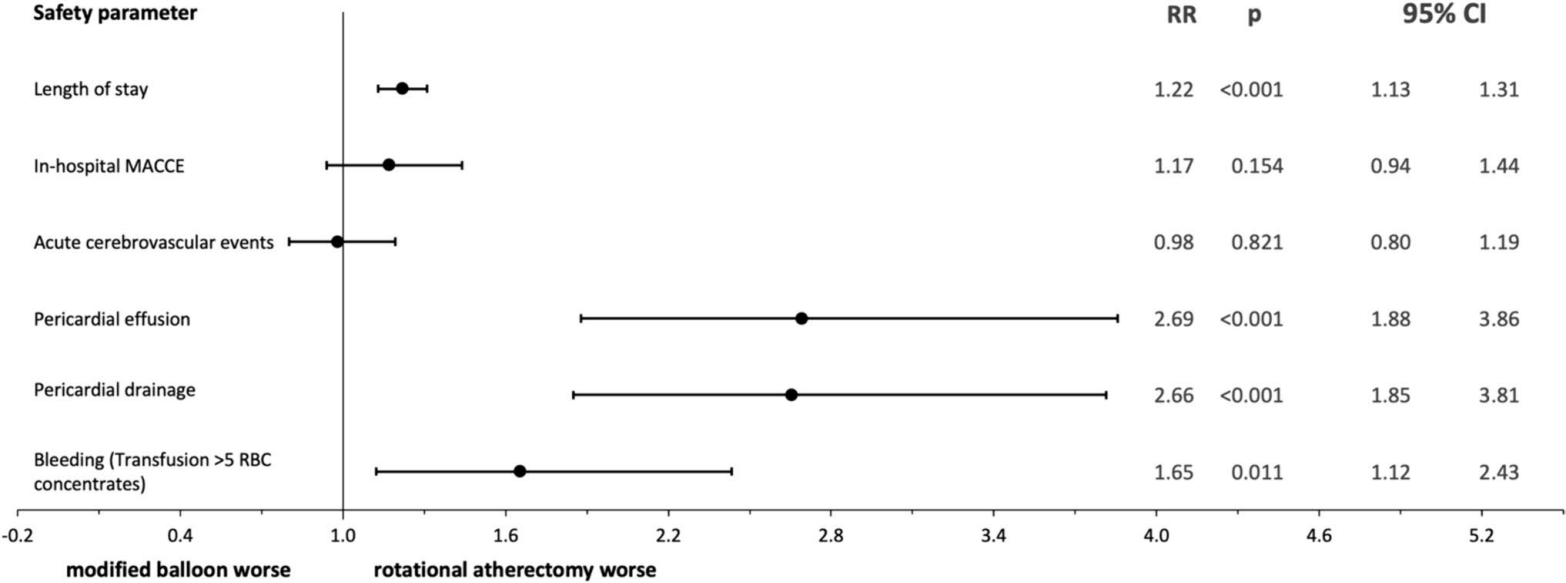
Forest plot of propensity score approach to compare adjusted risks of in-hospital safety parameters. CI: confidence interval, MACCE: major adverse cardiac and cerebrovascular events, RBC: red blood cell concentrate. For the propensity score estimation, we fit a logistic regression model controlling for 30 predetermined covariates (all variables listed in Table 1)

Total and relative in-hospital safety parameters for RA and MB angioplasty are given in Table 2. To compare the two calcified lesion treatment tools, we used inverse probability weighted analyses (Fig. 2). Adjusted risk of in-hospital MACCE and acute cerebrovascular events was similar for both procedures. After RA patients spent significantly more days in the hospital compared to MB angioplasty (6.41 ± 7.9 vs. 5.09 ± 7.3, *p* < 0.001; RR 1.22 95% CI 1.13–1.31; *p* < 0.001). Transfusion of more than five red blood cell concentrates (RBC) was observed significantly more often in patients receiving RA (0.79% vs 0.55%, *p* = 0.009; RR 1.65 95% CI 1.12–2.43%; *p* < 0.011). Further, risk of pericardial effusion (1.92% vs. 0.93%, *p* < 0.001; RR 2.69 95% CI 1.88–3.86; p < 0.001) as well as risk of pericardial drainage (1.03% vs. 0.41%, *p* < 0.001; RR 2.66 95% CI 1.85–3.81; *p* < 0.001) were significantly higher after RA. Importantly, in an additional analysis excluding 1,179 patients that were treated with both modified balloon and rotational atherectomy, these findings remained consistent (Supplemental Fig. 1).

**Table 2:** In-hospital outcome parameters for rotational atherectomy and modified balloon procedures in Germany between 2017 and 2020

Outcomes	Rotational atherectomy *n* = 10,092	modified balloon *n* = 22,378	*p*
Length of stay (days)	*6.41* ± *7.9*	5.09 ± 7.3	** < *0.001***
Stent implantation	*9,709 (96.20%)*	18,149 (81.10%)	** < *0.001***
Number of stents per patient	*2.31* ± *1.31*	1.60 ± 1.32	** < *0.001***
MACCE	*228 (2.26%)*	370 (1.65%)	** < *0.001***
Acute cerebrovascular events	502 (4.97%)	1,046 (4.67%)	0.240
Pericardial effusion	*194 (1.92%)*	208 (0.93%)	** < *0.001***
Pericardial drainage	*104 (1.03%)*	91 (0.41%)	** < *0.001***
Bleeding (Transfusion > five RBC concentrates)	*80 (0.79%)*	122 (0.55%)	***0.009***
Deep vein thrombosis	50 (0.50%)	96 (0.43%)	0.408

*MACCE*  in-hospital mortality/stroke/myocardial infarction, *Pericardial drainage* pericardial tamponade/pericardial puncture/pericardiotomy, *RBC* red blood cell

### Patient characteristic risk drivers and center-volume-dependent outcomes of rotational atherectomy

Since we observed more pericardial effusion, pericardial drainage and bleeding events in patients with RA, we next performed a risk-adjusted Poisson-regression analysis in order to identify the drivers in baseline characteristics of these risks. Through this analysis, we found NYHA III/IV (*p* < 0.001; RR 4.24; 95% CI 2.10–8.54) and atrial fibrillation (*p* < 0.001; RR 1.82; 95% CI 1.21–2.75) to be associated with a significantly higher risk of pericardial effusion (Supplemental Table 1). For pericardial drainage we found similar results with a higher risk in patients with NYHA III/IV (*p* < 0.001; RR 4.28; 95% CI 2.13–8.60) and atrial fibrillation (*p* < 0.001; RR 1.86; 95% CI 1.24–2.78, Supplemental Table 2). Risk of bleeding was higher in patients with NYHA III/IV (p < 0.001; RR 4.18; 95% CI: 1.93–9.07), atrial fibrillation (*p* = 0.005; RR 1.86; 95% CI 1.2–2.88)), in-stent stenosis (*p* = 0.048; RR 2.22; 95% CI: 1.01–4.88), mild liver disease (*p* = 0.004; RR 2.99; 95% CI: 1.41–6.33) and peptic ulcer disease (*p* < 0.001; RR 9.08; 95% CI: 3.90–21.14). Interestingly, in patients with chronic renal disease risk of bleeding was decreased (*p* = 0.007; RR 0.41; 95% CI: 0.21–0.78, Supplemental Table 3).

**Fig. 3:**
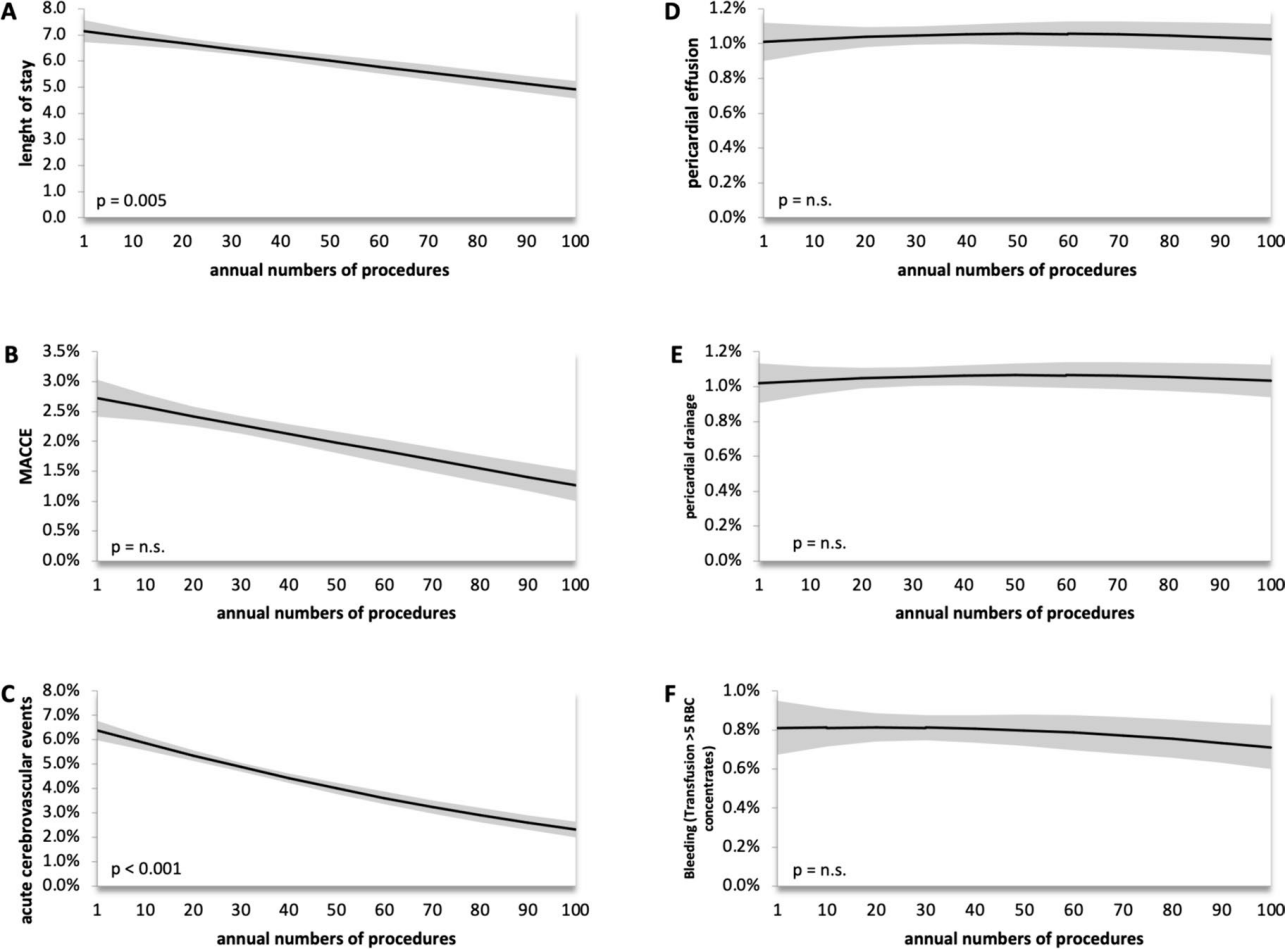
Rotational atherectomy center-volume-dependent outcomes

We in addition compared whether the number of RA procedures performed by a single center was associated with in-hospital outcomes. For pericardial effusion, pericardial puncture and bleeding we did not find a significant dependency on center volumes. Interestingly, we could observe an inverse association of the single-center annual procedure numbers with the length of hospitalization (*p* = 0.005) and acute cerebrovascular events (*p* < 0.001). MACCE was not influenced significantly by the annual procedure number of a single center (Fig. 3).

## Discussion

In our study we evaluated in-hospital outcomes of 32,470 patients undergoing RA or MB angioplasty in the German nationwide cohort from 2017 to 2020. Overall, patients treated by RA had a higher prevalence of risk factors associated with CAC such as male sex, more advanced age, chronic renal disease, and diabetes which can be expected since RA is reported to be used and recommended in lesions with a higher severity of calcification compared to MB angioplasty [25].

While MB angioplasty was performed more frequently than RA in general, annual procedure numbers increased for both interventions in our 4-year observational period. Similarly, a previous report of the National Cardiovascular Data CathPCI Registry analyzing the utilization of coronary atherectomy from 2009 to 2016 found that the numbers of coronary atherectomy has increased over time [26]. These developments may be driven by recent technical optimizations of the RA system [8, 27], the increasing prevalence of coronary calcifications [28] and the increasing confidence of interventionalists in attempting more challenging coronary interventions with the advent of new and improvement of established interventional tools [9].

With regards of in-hospital outcomes of RA and MB angioplasty, we found that the risk of pericardial effusion as well as the adjusted risk of necessity for pericardial puncture was significantly higher in patients undergoing RA. Further, transfusion of more than 5 RBC concentrates was also observed significantly more often in patients receiving RA, suggestive of increased frequency of relevant blood loss. These findings are somewhat expected as RA in general represents the more invasive technique and its use particularly in severely tortuous vessels goes alongside with the risk of coronary perforation [29]. Historically, RA treatment during PCI has been found to be associated with an increased risk of complications, with reported procedural complication rates up to 9.7% [30]. Notably these observations were confounded by a higher preprocedural risk profile of patients treated with RA. Also, increasing operator experience and further technical optimizations such as utilizing smaller burr sizes with a burr/artery ratio of 0.5 and current recommendations to employ shorter burring episodes likely contributes to lower peri-procedural complications in contemporary RA inteventions [29, 31, 32]. Evidence from recent randomized clinical trials emphasized that the use of RA, compared with cutting/scoring balloon-based strategies, is superior in terms of procedural success and not associated with increased intraprocedural or periprocedural complication rates [18, 21]. Notably, in PREPARE-CALC, pericardial effusions were numerically more frequent in patients after RA although these findings did not reach statistical significance like in our real-world study [21]. In line with recent trials, in-hospital adverse cardiovascular events in our study did no differ significantly for MB angioplasty and RA [18, 21].

The superior success rate of RA lesion preparation may contribute to the here observed higher number of implanted stents.

Notably, patients treated with RA spent significantly more days in hospital compared to cutting/scoring balloon angioplasty. While this to some extent may reflect pericardial complications and their management, this may also be explained by a greater disease burden in the overall sicker and older study population of the RA group. Moreover, due to its high technical success rate, RA is frequently used as a bail-out tool in complex coronary diseases, which make up to 20% of calcified coronary lesions in RCTs [18]. Even though recent evidence suggests that unplanned RA is generally associated with favorable outcome when compared to planned RA [33], interventions that involve crossing-over to RA to enable procedural success may require longer procedural time and more frequently demand management of periprocedural complications, which may lead to an increase in hospitalization durations [34, 35]. A total of 1179 cases employed MBs and RA during one hospital stay and thus likely represent such bail-out scenarios.

Finally, we aimed to identify patient characteristic risk drivers and center-volume-dependent outcomes of RA. In our analysis, the risk for the occurrence of pericardial effusion, pericardial drainage and bleeding events was significantly higher in cardiac patients with NYHA III/IV staged heart failure and atrial fibrillation. These findings are in line with others in the literature. Patients with atrial fibrillation undergoing percutaneous coronary intervention are typically at a higher risk of bleeding events due to the need for oral anticoagulation on top of antiplatelet therapy [36]. Further, heart failure has been identified as one of the major risk drivers of bleeding in previous studies [34–36]. While pericardial effusion itself is a common baseline finding in patients with congestive heart failure [37], impaired LVEF has also been associated with unfavorable in-hospital outcomes after PCI by several studies [38, 39]. Interestingly, we could observe an inverse association of annual procedure numbers with the length of hospitalization and the occurrence of acute cerebrovascular events. In a nationwide retrospective cohort study with 9970 patients from Japan a higher hospital volume of ≥ 31 RAs/year was significantly associated with lower complication rates of RA [40]. In line, recent registry data indicated a higher volume of coronary atherectomy procedures was associated with a lower risk of major adverse events but associated with a small increase in risk of coronary perforation [26]. In our study, we did not find a significant influence of the center volume on the risk of pericardial effusions or the necessity for pericardial drainage indicating that this risk may be attributed to the RA technique and the complexity of CAC itself rather than being dependent on interventionalists experience.

### Study limitations

Our study has several limitations as previous studies conducted with similar datasets [22, 23]. First, the ICD discharge code based data collection used for our analysis is potentially biased by under- or overreporting. However, it is very unlikely that the here used endpoints like in-hospital MACCE were under-reported, as an increase in resource-utilization often triggers an additional reimbursement. Second, it is not reported at what time of the hospital stay an outcome event occurred or if it’s associated with a procedure other than treatment of calcified coronary lesions of the same hospital stay. Third, we cannot address long-term outcomes with ICD discharge codes. Fourth, we do not have data on the presence or degree of coronary calcification; therefore, we cannot make any inferences about the appropriate or inappropriate use of RA or scoring/cutting balloons. Fifth, patients with acute coronary syndromes were excluded from our analysis we therefore cannot make any conclusions on the outcomes of RA and MBs in patients with myocardial infarction. Sixth, we cannot distinguish between scoring and cutting balloons in our analysis and can thus not assess differences between these subtypes of modified balloons which may differ in parameters such as crossability [41] and interventional success rate [42]. Last, coronary stenoses that are not crossable with a microcatheter or display deep calcium formations in the plaque or vessel wall on intravascular imaging may preferably be treated by excimer laser coronary angioplasty or intravascular lithotripsy, respectively [9, 43], which is not part of this dataset.

## Conclusions

Both RA and MBs are commonly and increasingly used procedures for calcified lesions in Germany. Patients receiving RA were older, had more comorbidities including more severe coronary artery disease compared to patients treated with MBs. Procedural safety regarding bleeding and the occurrence of pericardial effusion is superior for MBs. In centers with high annual rotational atherectomv procedure numbers the risk of acute cerebrovascular events is lower and the length of hospital stay is shorter.

## Supplementary Information

Below is the link to the electronic supplementary material.

Supplementary file1 (DOCX 156 KB)

## Data Availability

The data underlying this article are available in the article and in its online supplementary material.

## Abbreviations

CAC: Coronary artery calfication
CAD: Coronary artery disease
CI: Confidence interval
COPD: Chronic obstructive pulmonary disease
DES: Drug eluting stent
EAPCI: European association of percutaneous cardiovascular interventions
RBC: Red blood cell
MACCE: Major adverse cardiac and cerebrovascular events
MB: Modified balloon
NYHA: New York heart association
PCI: Percutaneous coronary intervention
RA: Rotational atherectomy
