# Supplementary material for: Procedural safety of rotational atherectomy and modified balloon angioplasty: insights from a German national registry
Source: Clin Res Cardiol. 2024 Sep 11;115(1):16–24. doi: 10.1007/s00392-024-02538-8 (PMC12783188; doi:10.1007/s00392-024-02538-8)
Supplement: Supplementary file 1 — Supplementary file1 (DOCX 156 KB) [file 392_2024_2538_MOESM1_ESM.docx]

**
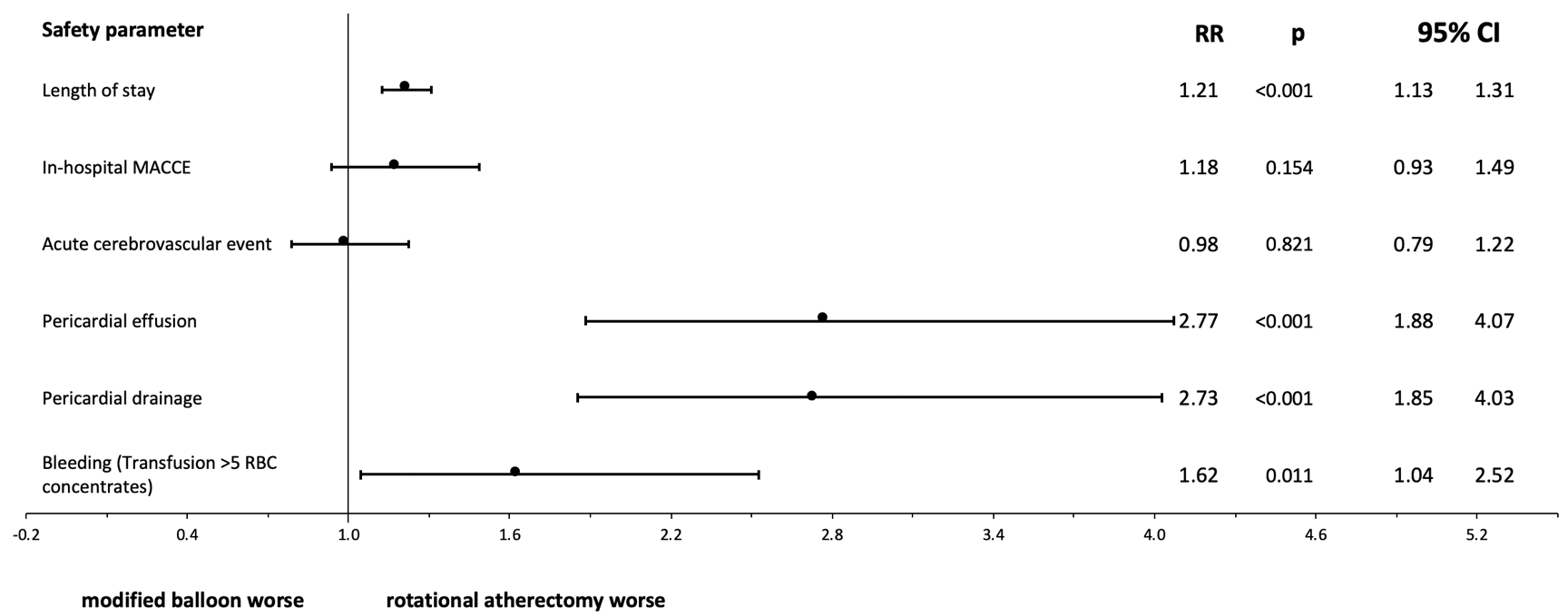
Supplemental Figure 1.** Forest plot of propensity score approach to compare adjusted risks of in-hospital safety parameters excluding patients treated by both RA and MB.

CI: confidence interval, MACCE: major adverse cardiac and cerebrovascular events, RBC: red blood cell concentrate. For the propensity score estimation, we fit a logistic regression model controlling for 30 predetermined covariates (all variables listed in Table 1). Patients which were treated by both, modified balloon and rotational atherectomy were excluded from this analysis

**Supplemental** Table **1.** Risk adjusted Poisson-regression analysis for pericardial effusion in rotational atherectomy.

| **Characteristic** | **RR** | **95% CI** | **p** |
| --- | --- | --- | --- |
| Women | 1.49 | 0.76 - 2.95 | 0.248 |
| Age | 1.01 | 0.96 - 1.07 | 0.685 |
| Charlson Comorbidity Index (CCI) | 1.00 | 0.72 - 1.38 | 0.997 |
| NYHA III or IV | *4.24* | *2.10 - 8.54* | ***<0.001*** |
| Arterial hypertension | 0.71 | 0.30 - 1.68 | 0.436 |
| Coronary artery disease | 0.48 | 0.04 - 5.45 | 0.552 |
| Coronary 1-vessel disease | 0.66 | 0.17 - 2.62 | 0.554 |
| Coronary 2-vessel disease | 1.25 | 0.36 - 4.28 | 0.723 |
| Coronary 3-vessel disease | 1.00 | 0.28 - 3.56 | 0.999 |
| Left main disease | 1.37 | 0.88 - 2.13 | 0.160 |
| In-stent stenosis | 1.34 | 0.55 - 3.25 | 0.521 |
| Previous myocardial infarction | 0.65 | 0.33 - 1.28 | 0.212 |
| Previous cardiac surgery | 0.58 | 0.29 - 1.17 | 0.126 |
| Atrial fibrillation | *1.82* | *1.21 - 2.75* | ***0.004*** |
| CHA_2_DS_2_ VASC | 1.02 | 0.54 - 1.92 | 0.958 |
| Peripheral vascular disease | 0.67 | 0.29 - 1.55 | 0.353 |
| Carotid disease | 0.77 | 0.25 - 2.39 | 0.647 |
| COPD | 1.09 | 0.49 - 2.40 | 0.832 |
| Pulmonary hypertension | 0.91 | 0.45 - 1.84 | 0.797 |
| Chronic renal disease | 1.08 | 0.52 - 2.23 | 0.845 |
| Diabetes | 0.62 | 0.23 - 1.67 | 0.343 |
| Previous stroke | 2.42 | 0.47 - 12.29 | 0.288 |
| Hemiplegia or paraplegia | *0.00* | *0.00 - 0.00* | ***<0.001*** |
| Dementia | 0.67 | 0.09 – 4.91 | 0.697 |
| Connective tissue disease | 0.88 | 0.12 - 6.71 | 0.906 |
| Peptic ulcer disease | *0.00* | *0.00 - 0.00* | ***<0.001*** |
| Mild liver disease | 0.68 | 0.09 - 5.05 | 0.709 |
| Moderate to severe liver disease | *0.00* | *0.00 - 0.00* | ***<0.001*** |
| Cancer | 1.29 | 0.29 - 5.68 | 0.737 |
| Metastatic solid tumor | *0.00* | *0.00 - 0.00* | ***<0.001*** |

**Supplemental** Table **2.** Risk adjusted Poisson-regression analysis for pericardial drainage in rotational atherectomy

| **Characteristic** | **RR** | **95% CI** | **p** |
| --- | --- | --- | --- |
| NYHA III or IV | *4.28* | *2.13 - 8.60* | ***<0.001*** |
| Arterial hypertension | 0.69 | 0.29 - 1.65 | 0.401 |
| Coronary artery disease | 0.47 | 0.04 - 5.35 | 0.543 |
| Voronary 1-vessel disease | 0.65 | 0.16 - 2.57 | 0.538 |
| Voronary 2-vessel disease | 1.23 | 0.36 - 4.18 | 0.742 |
| Coronary 3-vessel disease | 1.00 | 0.28 - 3.54 | 0.995 |
| Left main disease | 1.35 | 0.87 - 2.08 | 0.181 |
| In-stent stenosis | 1.33 | 0.55 - 3.24 | 0.532 |
| Previous myocardial infarction | 0.66 | 0.34 - 1.30 | 0.229 |
| Previous cardiac surgery | 0.57 | 0.28 - 1.15 | 0.118 |
| Atrial fibrillation | *1.86* | *1.24 - 2.78* | ***0.003*** |
| CHA_2_DS_2_ VASC | 1.06 | 0.55 - 2.03 | 0.865 |
| Peripheral vascular disease | 0.67 | 0.29 - 1.54 | 0.340 |
| Carotid disease | 1.05 | 0.39 - 2.80 | 0.928 |
| COPD | 1.09 | 0.49 - 2.39 | 0.837 |
| Pulmonary hypertension | 1.01 | 0.53 - 1.95 | 0.970 |
| Chronic renal disease | 1.09 | 0.53 - 2.26 | 0.814 |
| Diabetes | 0.64 | 0.24 - 1.66 | 0.355 |
| Previous stroke | 2.21 | 0.42 - 11.54 | 0.346 |
| Hemiplegia or paraplegia | *0.00* | *0.00 - 0.00* | ***<0.001*** |
| Dementia | 0.68 | 0.09 - 4.98 | 0.706 |
| Connective tissue disease | 0.89 | 0.12 - 6.72 | 0.912 |
| Peptic ulcer disease | *0.00* | *0.00 - 0.00* | ***<0.001*** |
| Mild liver disease | 0.70 | 0.10 - 5.18 | 0.729 |
| Moderate to severe liver disease | *0.00* | *0.00 - 0.00* | ***<0.001*** |
| Cancer | 1.34 | 0.31 - 5.87 | 0.696 |
| Metastatic solid tumor | *0.00* | *0.00 - 0.00* | ***<0.001*** |

**Supplemental Table 3.** Risk adjusted Poisson-regression analysis for bleeding in rotational atherectomy.

| **Characteristic** | **RR** | **95% CI** | **p** |
| --- | --- | --- | --- |
| Women | 1.67 | 0.70 - 3.99 | 0.250 |
| Age | 0.97 | 0.91 - 1.03 | 0.343 |
| Charlson Comorbidity Index (CCI) | *1.39* | *1.07 - 1.80* | ***0.015*** |
| NYHA III or IV | *4.18* | *1.93 - 9.07* | ***<0.001*** |
| Arterial Hypertension | 0.44 | 0.19 - 1.03 | 0.060 |
| Coronary artery disease | 0.32 | 0.05 - 2.09 | 0.232 |
| coronary 1-vessel disease | 0.39 | 0.08 - 1.79 | 0.224 |
| coronary 2-vessel disease | 0.42 | 0.12 - 1.52 | 0.187 |
| coronary 3-vessel disease | 0.55 | 0.16 - 1.85 | 0.335 |
| left main disease | 1.21 | 0.67 - 2.19 | 0.526 |
| In-stent stenosis | *2.22* | *1.01 - 4.88* | ***0.048*** |
| Previous myocardial infarction | 0.65 | 0.35 - 1.20 | 0.169 |
| Previous cardiac surgery | 1.01 | 0.51 - 1.98 | 0.984 |
| Atrial fibrillation | 1.86 | 1.20 - 2.88 | 0.005 |
| CHA_2_DS_2_ VASC | 1.29 | 0.62 - 2.68 | 0.490 |
| Peripheral vascular disease | 1.58 | 0.94 - 2.66 | 0.086 |
| Carotid disease | 0.82 | 0.32 - 2.12 | 0.687 |
| COPD | 0.59 | 0.26 - 1.36 | 0.214 |
| Pulmonary hypertension | 1.61 | 0.86 - 3.01 | 0.138 |
| Chronic renal disease | *0.41* | *0.21 - 0.78* | ***0.007*** |
| Diabetes | 0.58 | 0.26 - 1.29 | 0.184 |
| Previous Stroke | 0.31 | 0.02 - 4.16 | 0.378 |
| Hemiplegia or paraplegia | *0.00* | *0.00 - 0.00* | ***<0.001*** |
| Dementia | 0.87 | 0.16 - 4.82 | 0.875 |
| Connective tissue disease | 0.77 | 0.10 - 5.96 | 0.799 |
| Peptic ulcer disease | *9.08* | *3.90 - 21.14* | ***<0.001*** |
| Mild liver disease | *2.99* | *1.41 - 6.33* | ***0.004*** |
| Moderate to severe liver disease | 0.98 | 0.11 - 9.02 | 0.987 |
| Cancer | 0.72 | 0.16 - 3.32 | 0.675 |
| Metastatic solid tumor | *0.00* | *0.00 - 0.00* | ***<0.001*** |
